# miRNA expression and function in thyroid carcinomas: a comparative and critical analysis and a model for other cancers

**DOI:** 10.18632/oncotarget.9655

**Published:** 2016-05-28

**Authors:** Manuel Saiselet, Jaime M. Pita, Alice Augenlicht, Geneviève Dom, Maxime Tarabichi, Danai Fimereli, Jacques E. Dumont, Vincent Detours, Carine Maenhaut

**Affiliations:** ^1^ Institute of Interdisciplinary Research (IRIBHM), University of Brussels, Brussels, Belgium; ^2^ WELBIO, School of Medicine, University of Brussels, Brussels, Belgium

**Keywords:** miRNA, cancer, function, expression profile, thyroid

## Abstract

As in many cancer types, miRNA expression profiles and functions have become an important field of research on non-medullary thyroid carcinomas, the most common endocrine cancers. This could lead to the establishment of new diagnostic tests and new cancer therapies. However, different studies showed important variations in their research strategies and results. In addition, the action of miRNAs is poorly considered as a whole because of the use of underlying dogmatic truncated concepts. These lead to discrepancies and limits rarely considered. Recently, this field has been enlarged by new miRNA functional and expression studies. Moreover, studies using next generation sequencing give a new view of general miRNA differential expression profiles of papillary thyroid carcinoma. We analyzed in detail this literature from both physiological and differential expression points of view. Based on explicit examples, we reviewed the progresses but also the discrepancies and limits trying to provide a critical approach of where this literature may lead. We also provide recommendations for future studies. The conclusions of this systematic analysis could be extended to other cancer types.

## INTRODUCTION

MicroRNAs are a class of small non-coding RNAs, 19 to 25 nucleotides long, which repress the stability and the translation efficiency of their target mRNAs [1]. Their expression levels may classify different histological and pathological types of human tissues [2]. miRNAs have become major recognized actors in molecular cell biology in general and in cancer physiopathology and diagnosis in particular [3]. It has been suggested that changes in the expression of multiple miRNAs could be a major mechanism in thyroid cancer tumorigenesis [4] and could be used in diagnosis [5]. miRNA studies represent now an important chapter in thyroid cancer research.

Thyroid cancer is the most frequent cancer of the endocrine glands and the fifth most frequent cancer in women, with an increasing incidence [6]. It represents a useful model for other cancers because its various types present distinct histological features and the evolution from one type to another is rather well defined. Human thyroid tumors may derive from follicular (thyrocytes) or parafollicular C cells. Medullary thyroid cancer is the only form of tumor derived from parafollicular C cells. They represent a very small part of all thyroid tumors (2-4%) [7, 8]. On the other hand, different types of thyroid tumors derive from follicular cells. Each type is characterized by distinct molecular, clinical and histological criteria. Two types of benign tumor are described: autonomous adenoma (AA) and follicular adenoma (FA). They are characterized by their respectively high and low capacity to take up iodide and produce thyroid hormones. Three types of thyroid carcinoma are described: well-differentiated, poorly differentiated and undifferentiated or anaplastic carcinoma (ATC). The well-differentiated type may be subdivided into two subtypes: papillary thyroid carcinoma (PTC) and follicular thyroid carcinoma (FTC). PTC is the most frequent type of thyroid cancer (80-85%) and originates de novo while FTC (10-15%) may derive from FA or directly from normal follicular cells [7, 8]. The long term survival of both tumors is good (greater than 80% at 10 years) and both present a loss of differentiation with decreased capacity to take up iodide and decreased expression of specific differentiation markers (e.g. TSH receptor) [7, 8]. ATC is one of the most aggressive human tumors; the thyrocytes are completely dedifferentiated and nonfunctional and the one year survival rate is estimated at 10 to 20% [9]. This tumor either derives from differentiated tumors or directly from normal follicular cells. However, ATC represents only 1 to 3% of all thyroid tumors [7–9].

Great progress has been made in the diagnosis and the understanding of the physiopathology of human non-medullary thyroid carcinomas. Nevertheless, the nature of the transition from a slowly evolving differentiated adenoma to a highly aggressive anaplastic carcinoma remains poorly understood. In addition, the establishment of a molecular signature that can increase the accuracy of the cancer diagnosis on thyroid biopsies is still an important subject of research [5, 10].

The most exhaustive and cited review on miRNAs in human thyroid cancer, by Pallante *et al*, [11] summarized data on miRNA expression variations compared to normal samples (miRNA differential expression profiles) in various thyroid cancers, published until 2013. As in other recent reviews [12], the results of the literature are reported in the form of tables with little comparative analyses of the different studies. Such analyses should lead the reader to a better understanding of the main conclusions but also limits and the discrepancies which are rarely considered. General miRNA differential expression profiles of non-medullary thyroid carcinomas are described but the results of different studies show strong variations. Even considering the variations in their experimental procedures (types of samples, quantity and repartition of samples, methodology and significance criteria used), it is difficult to draw simple conclusions from these syntheses. From such discrepant results some miRNAs are chosen to be integrated into functional schemes. From this analysis, diagnostic and therapeutic implications are derived and discussed. The miRNA expression data used by Pallante *et al.*, and authors of similar reviews, are qRT-PCR or miRNA microarrays data. Since then, the field has been improved by the release of new miRNA functional and expression profiling studies. Next generation sequencing (small RNA deep sequencing technologies) gives a new reading of miRNA differential expression profiles of PTC and FTC [13–19].

The aims and thus the strategies of miRNA expression studies in cancer may differ: they can either define general differential expression profiles and diagnostic signatures or define physiopathological mechanisms and possible therapeutic approaches [20]. In the present review, we shall analyze separately these two viewpoints by reviewing in detail progresses but also discrepancies in the literature of miRNAs in the non-medullary thyroid cancers field. PubMed referenced 290 articles on “miRNA and thyroid cancer” on October 2015. We aimed to provide a critical approach of where this literature may lead. The conclusions of this systematic analysis could be ultimately applied to other cancer types.

## DIFFERENTIAL EXPRESSION PROFILING

### General considerations

We performed a systematic review of the articles showing miRNA differential expression profiles between human thyroid carcinomas (PTC, FTC or ATC) and normal samples until end of October 2015. After exclusion of studies using cell lines or pooled samples, a total of 24 studies were kept for further analyses. We describe the research strategy used in each study regarding the number and the repartition of samples, the methodologies and the significance criteria used (Table 1–3). All these studies are difficult to compare as they differ in:

**Table 1:** synthesis of research strategies of general miRNA expression profiles studies of non-medullary thyroid carcinomas compared to normal tissues following literature analysis, until October 2015

References	First sample set	Methodology	Significance criteria	Independent validation set
He et al. 2005 [117]	15 PTC vs NPTC	μarrays (235)	SAM (qval=0)	NA
Pallante et al. 2006 [118]	30 PTC vs 10 NT	μarrays (245)	ANOVA, T-test (pval<0.05)	39 PTC vs NPTC
Tetzlaff et al. 2007 [119]	10 PTC vs 10 MNG	μarrays (219)	SAM (FDR<0.05)	10 PTC vs 10 MNG
Nikiforova et al. 2008 [40]	9 PTC vs 5 NT	qPCR panel (158)	T-test (FDR<0.05)	6 PTC vs 3 HN
Yip et al. 2011 [98]	12 PTC vs 4 NT	μarrays (319)	modulated in each sample	NA
Lassalle et al. 2011 [22]	16 PTC vs NPTC	μarrays (462)	log2 fold changes > |0.58|	NA
Huang et al. 2013 [29]	12 PTC vs 3 NT	μarrays (866)	SAM (FDR=0)	NA
Dettmer et al. 2013 [38]	44 PTC (>80%) vs 8 NT	qPCR panel (754)	MWW (pval<0.05)	46 PTC vs 5 NT
Zhang et al. 2013 [21]	3 PTC vs NPTC	μarrays (1090)	T-test (pval<0.05) log2 fold changes > |1|	NA
Jacques et al. 2013 [120]	2 PTC vs NPTC	μarrays (866)	T-test (pval<0.05)	5 PTC vs 5 NT
Wang et al. 2013 [121]	6 PTC vs 2 NT	μarrays (1205)	T-test (pval<0.05)	NA
Peng et al. 2014 [30]	8 PTC vs 4 MNG	μarrays (1223)	fold changes >2 or <0.5	NA
Swierniak et al. 2013 [13]	14 PTC vs NPTC	small RNA deep Seq	T-test (FDR<0.05)	9 PTC vs NPTC
TCGA 2014 [15]	495 PTC (>60%) vs 59 NT	small RNA deep Seq	RPM>50 SAMseq (FDR<0.05) Wilcoxon test (pval<0.05)	NA
Mancikova et al. 2015 [17]	35 PTC (>80%) vs 8 NT	small RNA deep Seq	edgeR (FDR<0.01) fold changes >2 or <0.5	43 PTC (>80%) vs 9 NT
Saiselet et al. 2015 [19]	3 PTC (>70%) vs NPTC	small RNA deep Seq	edgeR (FDR<0.05)	14 PTC (>70%) vs NPTC TCGA data
Riesco-Eizaguirre et al. 2015 [18]	8 PTC vs NPTC	small RNA deep Seq	RPM >0.6 edgeR (FDR<0.05) fold changes >1.5 or <0.66	16 PTC vs 14 NT 8 PTC vs 8 NT

We describe the composition of the first sample set analyzed, the quantification methodology and the statistical significance criteria used to analyze this first set and the composition of the independent validation set of samples used if provided. The methodology for the analysis of the independent validation set may be different from the one used for the first set, and was most of the time qRT-PCR. When mentioned in the original publication, the percentages of cancer cells considered to determine the inclusion or rejection of a tumor sample in the study are mentioned in brackets in the “First sample set” and in the “Independent validation set” columns. The numbers of human mature miRNAs analyzed by each microarray or quantitative PCR platform are mentioned in brackets in the “Methodology” column. PTC: papillary thyroid cancer; NPTC: associated normal tissue of papillary thyroid cancer; FTC: follicular thyroid cancer; NFTC: associated normal tissue of follicular thyroid cancer; ATC: anaplastic thyroid cancer; NT: normal thyroid tissue; MNG: multinodular goiter; HN: hyperplasic nodule; NA: no independent validation set in the study; SAM: significance analysis of microarrays; MWW: Mann–Whitney–Wilcoxon U test; FDR: false discovery rate; RPM: reads per millions.

**Table 2:** synthesis of research strategies of general miRNA expression profiles studies of non-medullary thyroid carcinomas compared to normal tissues following literature analysis, until October 2015

References	First sample set	Methodology	Significance criteria	Independent validation set
Nikiforova et al. 2008 [40]	5 FTC vs 5 NT	qPCR panel (158)	T-test (FDR<0.05)	4 FTC vs 3 HN
Reddi et al. 2011 [77]	12 FTC vs 7 NT	μarrays (1146)	mean expression differences ≥ 3 SD	10 FTC vs 3 NT
Lassalle et al. 2011 [22]	6 FTC vs NFTC	μarrays (462)	log2 fold changes > |0.58|	NA
Rossing et al. 2012 [122]	12 FTC vs 10 NT	μarrays (841)	T-test (FDR<0.05) fold changes >2 or <0.5	NA
Dettmer et al. 2013 [23]	38 FTC (>80%) vs 10 NT	qPCR panel (381)	T-test (FDR<0.05)	NA
Wojtas et al. 2014 [123]	10 FTC vs NFTC	μarrays (1146)	T-test (FDR<0.05) fold changes >2.5 or <0.4	11 FTC vs NFTC
Mancikova et al. 2015 [17]	17 FTC (>80%) vs 8 NT	small RNA deep Seq	edgeR (FDR<0.01), fold changes >2 or <0.5	6 (>80%) FTC vs 9 NT

We describe the composition of the first sample set analyzed, the quantification methodology and the statistical significance criteria used to analyze this first set and the composition of the independent validation set of samples used if provided. The methodology for the analysis of the independent validation set may be different from the one used for the first set, and was most of the time qRT-PCR. When mentioned in the original publication, the percentages of cancer cells considered to determine the inclusion or rejection of a tumor sample in the study are mentioned in brackets in the “First sample set” and in the “Independent validation set” columns. The numbers of human mature miRNAs analyzed by each microarray or quantitative PCR platform are mentioned in brackets in the “Methodology” column. PTC: papillary thyroid cancer; NPTC: associated normal tissue of papillary thyroid cancer; FTC: follicular thyroid cancer; NFTC: associated normal tissue of follicular thyroid cancer; ATC: anaplastic thyroid cancer; NT: normal thyroid tissue; MNG: multinodular goiter; HN: hyperplasic nodule; NA: no independent validation set in the study; SAM: significance analysis of microarrays; MWW: Mann–Whitney–Wilcoxon U test; FDR: false discovery rate; RPM: reads per millions.

**Table 3:** synthesis of research strategies of general miRNA expression profiles studies of non-medullary thyroid carcinomas compared to normal tissues following literature analysis, until October 2015

References	First sample set	Methodology	Significance criteria	Independent validation set
Visone et al. 2007 [24]	10 ATC vs 10 NT	μarrays (161)	T-test (pval<0.05)	20 ATC vs 20 NT
Nikiforova et al. 2008 [40]	2 ATC vs 5 NT	qPCR panel (158)	T-test (FDR<0.05)	2 ATC vs 3 HN
Braun et al. 2010 [101]	3 ATC vs 3 NT	μarrays (773)	fold change of each tumor/normal pair >2 or <0.5	NA
Hebrant et al. 2014 [53]	11 ATC vs 19 NT	μarrays (841)	fold change of each tumor >1.5 or <0.66	NA

We describe the composition of the first sample set analyzed, the quantification methodology and the statistical significance criteria used to analyze this first set and the composition of the independent validation set of samples used if provided. The methodology for the analysis of the independent validation set may be different from the one used for the first set, and was most of the time qRT-PCR. When mentioned in the original publication, the percentages of cancer cells considered to determine the inclusion or rejection of a tumor sample in the study are mentioned in brackets in the “First sample set” and in the “Independent validation set” columns. The numbers of human mature miRNAs analyzed by each microarray or quantitative PCR platform are mentioned in brackets in the “Methodology” column. PTC: papillary thyroid cancer; NPTC: associated normal tissue of papillary thyroid cancer; FTC: follicular thyroid cancer; NFTC: associated normal tissue of follicular thyroid cancer; ATC: anaplastic thyroid cancer; NT: normal thyroid tissue; MNG: multinodular goiter; HN: hyperplasic nodule; NA: no independent validation set in the study; SAM: significance analysis of microarrays; MWW: Mann–Whitney–Wilcoxon U test; FDR: false discovery rate; RPM: reads per millions.

#### The number of patients and the population selection bias

The number of patients is a well-known source of variation between studies. It is only recently that a large cohort of 495 PTC samples has been analyzed [15], but with a relatively limited number of available normal samples (59). A variable number of samples was analyzed in the PTC studies, with a minimum of three tumors and normal samples [21]. This makes the direct comparison of the results of the different studies complicated (Table 1). The same observation can be done for the 7 FTC studies described. A variable number of samples was analyzed: from 6 tumors and normal samples [22] to 38 tumors and 10 normal samples [23] (Table 2). The very limited prevalence of ATC in the general population challenges the collection of a large cohort of samples. Indeed, the largest study analyzed 30 tumors and normal samples [24] while the 3 others analyzed fewer samples (Table 3). Moreover, it is important to know that most of the studies on miRNA thyroid tumors expression profiles concerned Caucasian populations. Recently, a preliminary study showed that some variations may appear between the miRNA expression profiles of PTC samples from Caucasian American patients and African American patients but these results require further confirmations regarding possible confounding factors [25].

#### The quantification method used

Three methods are mostly used, namely miRNA microarray, qRT-PCR and small RNA deep sequencing. A meta-analysis of published microarray data confirmed by qRT-PCR on 76 pathological samples validated 2 miRNAs for a potential signature of FTC against FA [26]. However, a further analysis by small RNA deep sequencing by the same authors showed different results [14]. The recent small RNA deep sequencing technology, thanks to its high resolution, can outperform miRNA microarray and qRT-PCR, by providing a more accurate quantification based on the detection of each miRNA isoform (isomiR) independently. In addition, it allows the identification of single base variations, including individual single nucleotide polymorphisms, mutations, RNA-editing and 3′non-templated nucleotide additions [27, 28]. The constant discovery of new miRNAs and the development of new miRNA expression profiling platforms created significant variations in the number of miRNAs analyzed in the 24 reported studies (Table 1-3). The majority of the studies have used microarrays analyzing from 161 up to 1223 different miRNAs. On the other hand, the use of small RNA deep sequencing in 5 studies allowed the analysis of all known miRNAs and the potential discovery of new miRNAs. Consequently, the discrepant results between studies could be due to the absence of specific miRNAs in some profiling platforms. miR-204-3p and miR-7-3p are examples of such miRNAs: their expressions have been reported as decreased in PTC in recent small RNA deep sequencing studies [18, 19] but these miRNAs are absent from the microarray platform used in a study using similar samples in 2011 [22]. Actually, miR-204-3p is still missing in more recent microarrays platforms [29, 30].

#### The use of a validation sample set and the criteria of statistical significance

It is advisable to use well-confirmed statistical analyses with commonly used significance criteria and two independent sets of samples in order to assert the accuracy and the comparability of an expression profile. The modulation of each miRNA must be defined on a training set and validated on another set of samples by a similar or a different quantification methodology. Those sets should be independent and as similar as possible. However, only 12 of the 24 referenced studies used a completely independent validation set of samples (Table 1-3). Additionally, the statistical tests used and the associated significance criteria can drastically change the results of a study. Regarding this matter, the 24 studies showed poorly homogenous protocols. The student *t*-test was commonly used and generalizations of this test in tools applied to microarrays (e.g. Significance Analysis of Microarrays) or deep sequencing technologies (edgeR) were used in 18 of the 24 studies but only in 2 of the 4 ATC studies. Moreover, the required correction for multiple testing was only used in 13 of the 18 studies including one of the 4 ATC studies. Furthermore, 10 of the 24 studies used fold change thresholds to limit the results to the most interesting miRNA modulations in terms of cancer physiology (Table 1-3).

#### The heterogeneity of the tumors and the cell contamination

The heterogeneity of the tumors could lead to different expression results for different samples of the same tumor type or for different parts of the same tumor [31] but this is rarely investigated. Single cell genome sequencing reveals high heterogeneity from cell to cell. This casts doubts on the general validity of global quantification on pieces of tumor tissues [32, 33]. Moreover, miRNA expression modulations in tumor samples might reflect a dilution of cancer cells by stromal cells (e.g. lymphocytes, fibroblasts) [34, 35]. On the other hand, changes in a subpopulation of cells (e.g. stromal cells) may be relevant to the physiopathology of cancer (e.g. breast cancer) [36] and a miRNA expression modulation nonspecific to cancer cells could actually be used in a clinical signature. Nevertheless, this will blur the basic research on the physiopathology of cancer cells. Unfortunately, on the 24 referenced studies only 5 used a threshold of cancer cells concentration in the tumor samples included, and this threshold varies from 60 to 80 % (Table 1-3).

### Description and analyses of the comparative tables

In accordance with the above considerations, it is not surprising that comparative results of the reported studies look rather confusing. The differences in the research strategy complicate the results of a meta-analysis that would be difficult to correctly interpret. Within this framework, we report the number of modulated miRNAs described in each study and exhaustive comparative lists of these miRNAs (supplementary file 1). The results are highly heterogeneous but we found several interesting observations:

#### miRNA expression profiles in PTC

A total of 125 up-regulated and 140 down-regulated miRNAs are reported but only a few are described in the majority of the studies. Some miRNAs are described as both down- and up-regulated in different studies (e.g. miR-195-5p). However, it is very interesting to observe that small RNA deep sequencing studies clearly changed the reported miRNA expression profile for this carcinoma. The 12 qRT-PCR or microarrays studies described a limited number of modulated miRNAs: from 5 to 25 up and from 0 to 11 down-regulated miRNAs with a great variation in the results. Only a few miRNA are found in at least three different studies. The over-expression of miR-146b-5p, miR-221-3p and miR-222-3p are reported in a majority of these studies. This led many authors to believe that PTC do not present common under-expressed miRNA and that these 3 miRNAs are the major modulated miRNAs in PTC (Table 4 and supplementary file 1). This concept was partially invalidated by the 5 recent small RNA deep-sequencing studies. These studies reported more modulated miRNAs: from 10 to 43 up- and from 5 to 77 down-regulated miRNAs. In addition, the majority of these studies reported 13 common up-regulations including miR-146b-5p, miR-221-3p and miR-222-3p and 17 down-regulations (e.g miR-7-3p, miR-204-5p, miR-1179) (Table 4 and supplementary file 1). These modulations have been mostly described in studies using an independent validation sample set analyzed with an alternative methodology (mostly qRT-PCR).

Follicular variants of PTC represent a challenging category of PTC. They show follicular patterns of cells but the cytology is related to papillary cancer [37]. Most of the time, they are analyzed together with classical forms and other uncommon variants. It has been shown that the miRNA expression profiles of follicular variants and classical variants are quite similar [15, 22, 38]. However, strong differences may be found in the intensity of modulations of some specific miRNAs [15, 19, 38]. Classical forms are the most frequent and are enriched by a specific mutation in the BRAF gene (BRAF V600E) while follicular variants present mutations in RAS genes or the BRAF K601E mutation. These mutations lead to different expression profiles in the tumor cells [8, 15]. The majority of commonly reported deregulated miRNAs in PTC by small RNA deep sequencing studies (Table 4) show an increase of their level of modulations according to the mutational status of the tumor. All the up-regulated miRNAs show increased expression in BRAF V600E positive tumor samples compared to BRAF V600E negative tumor samples, except miR-34a-5p, miR-182-5p and miR-183-5p. Similarly, the down-regulated miR-7-2-3p, miR-138-3, miR-138-5p, miR-139-5p, miR-152-3p, miR-204-5p, miR-652-3p, miR-873-5p and miR-1179 show decreased expression in BRAF V600E positive tumor samples compared to BRAF V600E negative tumor samples [15, 19].

#### miRNA expression profiles in FTC

A total of 41 up-regulated and 79 down-regulated miRNAs are reported but only few miRNAs are described in a majority of studies. These showed variable numbers of modulated miRNAs: from 0 to 26 up- and from 0 to 40 down-regulated miRNAs. Contrary to PTC, only one small RNA deep-sequencing study is available, which reported 26 up- and only 5 down-regulated miRNAs. miR-96-5p, miR-182-5p and miR-221-3p are the only overexpressed miRNAs consistently reported whereas no common under-expression is observed. Only the down-regulation of miR-31-5p, miR-199a-5p and miR-199b-5p are described in at least three different studies (Table 4 and supplementary file 1).

#### miRNA expression profiles in ATC

A qRT-PCR study reported 10 up-regulated miRNAs but no down-regulation. This observation was invalidated by 3 microarray studies showing that ATC present down-regulated miRNAs but very few up-regulations. In total, these 3 studies described 44 down-regulated miRNAs and only 6 up-regulated miRNAs. The under-expression of 13 miRNAs (e.g. miR-30d-5p, miR-141-3p, miR-200b-3p) are reported in at least 2 of these 3 studies. Decreased expression of the EMT regulators miR-30a-5p and miR-125b-5p are reported in the 3 studies and could be considered further in a diagnostic signature (Table 4 and supplementary file 1).

### Diagnostic miRNAs in FNAB and blood samples

Differential expression profiles may also be used on fine needle aspiration biopsies (FNAB), surgical materials or blood samples to diagnose and distinguish the different types of human thyroid cancer. One of the advantages of miRNAs is their stability in diagnostic material [39–42]. A large number of studies describe miRNA diagnostic signatures in thyroid FNAB and blood samples [5, 26, 43–49]. They present similar variations in their research strategies and results as in the studies on surgical materials.

Most of the studies on FNAB analyzed miRNAs that were previously described as modulated in tissue samples. The tested miRNAs were selected arbitrarily [43, 44, 47] or by meta-analysis of the previous data [26]. Variable diagnostic accuracies are described for each single miRNA qRT-PCR assay or for a combination of them. However, in a recent meta-analysis, Zhang *et al* [5] reported that multiple miRNA assays used together showed a higher diagnostic accuracy than single miRNA assays. Accordingly, Labourier *et al.* [47] recently showed that an expression signature of 10 miRNAs can increase the diagnostic accuracy of a previous test of malignancy on thyroid FNAB which is based on the detection of specific mutations. This could represent an alternative to recent ones based on DNA next generation sequencing technology [10].

Studies on blood samples are limited by the scarce knowledge on circulating miRNAs in the blood of thyroid cancer patients. So far, contradictory results have been obtained. miR-95-3p and miR-190-5p were proposed as diagnostic blood markers because they showed respectively increased and decreased expression in both tumor and blood samples of a cohort of Caucasian patients with PTC [49]. However, these miRNAs are different from those reported two years earlier in the blood samples of a Chinese cohort: increased expression of let-7e-5p, miR-151-5p and miR-222-3p [45]. This strong difference could be linked to a population selection bias. Nevertheless, the modulation of miR-222-3p has also been confirmed in the blood of Australian patients [46]. Very recently, another similar study performed in a Canadian cohort identified the up-regulated miR-10a-5p and let-7b-5p and the down-regulated miR146a-5p and miR-199b-5p as diagnostic blood markers of PTC. In this study, none of the previously cited miRNAs was reported as modulated [48]. Inconsistent results have been found also for other cancer types [50]. This could be explained by methodological pitfalls and limitations that are inherent to the screening of miRNAs in blood samples. Indeed, circulating miRNAs detected in serum or plasma samples may originate from tumors cells or other tissues. They are included in specific vesicles or released freely in the blood flow. However, blood cells also express miRNAs. Therefore, the miRNA expression profile is dependent on each pre-analytical step. For instance, the source of samples (serum *vs* plasma) and their processing, the miRNA extraction method or the presence of contaminating miRNAs originated from the lysis of blood cells dramatically influence the expression profiles [50–52]. Furthermore, other factors such as the normalization method used can influence the expression profiles, leading to a general lack of reproducibility. In our review of the literature, each study used its own methodology. For instance, only the Canadian study considered the potential hemolysis in the samples [48] while plasma samples were used only in the Australian study [46]. Further works with improved methodology are needed to clarify the situation.

### Conclusions, perspectives and remaining challenges

Even if the number of studies is limited, the small RNA deep sequencing studies gave a new view of the miRNA expression profile of PTC. We therefore believe that such studies designed with a thoughtful experimental strategy would be helpful to improve the miRNA expression profiles of FTC and ATC. So far, PTC and FTC present some common overexpressed miRNAs: miR-182-5p, miR-183-5p, miR-221-3p and miR-222-3p. ATC samples present a completely different expression profile (Table 4). This suggests that the progression of differentiated thyroid carcinomas to ATC is characterized by drastic changes in the miRNA expression profiles.

There is no doubt that, when used with a proven methodology, miRNA expression signatures can contribute to a satisfactory separation of thyroid carcinomas and benign nodules using FNAB samples. Newly described deregulated miRNAs by small RNA deep sequencing could increase the accuracy of diagnostic tests. However, the studies on circulating miRNAs in blood samples are still preliminary and present too many divergent results to conclude on their utility as diagnostic markers.

The establishment of differential expression profiles between FTC and FA and the early detection of poorly differentiated carcinoma and ATC remain as challenges. However, the latter carcinomas present a very low prevalence in many populations [6, 8].

## PHYSIOPATHOLOGY

### General considerations

**Figure 1:**
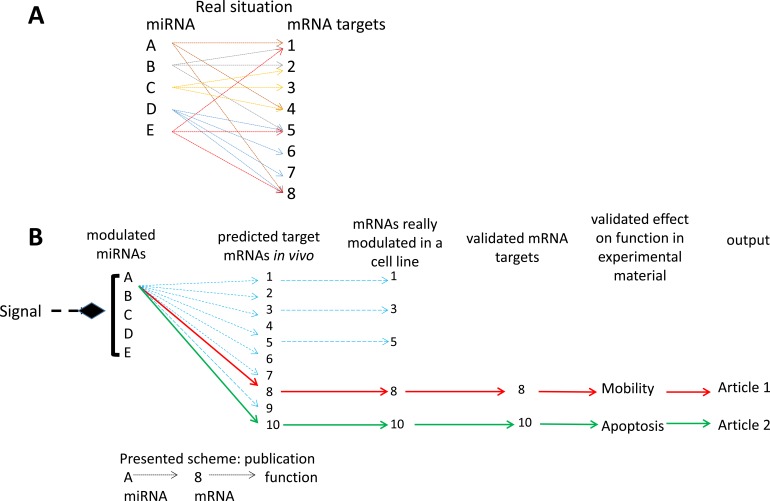
interrelations between miRNAs and their mRNA targets **A.** the bidirectional multiple controls model: several mRNA targets per miRNA and several controlling miRNAs per mRNA; **B.** the linear control model: one miRNA - one mRNA target- one biological effect, implicit in most miRNA functional studies.

In general, miRNAs are found to be differentially expressed in one type of carcinoma after a preliminary screening. Then, their modulations are confirmed by a specific experiment (e.g. qRT-PCR) in a larger series of samples. Their possible mRNA targets are predicted using one or more available bioinformatics algorithms. A screen of the literature and/or specific experiments point out the predicted mRNAs that are indeed regulated as expected in the carcinoma. A cell line is then searched in which the overexpressed miRNA represses its mRNA target and/or conversely, the repression of the miRNA enhances its target expression. This suggests a cell biology program (e.g. EMT, cell proliferation) that is shown to be altered in the right direction by miRNA and its siRNA counterpart *in vitro*. Potentially, *in vivo* confirmation experiments are conducted by injection of transfected cell lines subcutaneously in immunodeficient mice (Xenograft mouse model). Finally, a sequence from the miRNA expression to the pathology is proposed and the miRNA is suggested as a therapeutic target (Figure 1). The present stereotype is so prevalent that publications demonstrating striking changes in miRNA expression without a classical analysis of mRNA targets are difficult to publish. Our group has also participated in this same fashion [53]. Before exploring the comparative results, a short analysis of the accepted concepts of miRNAs action is in order.

#### Regulation of mRNA expression by miRNAs is a complex network

The control of gene expression by miRNAs may take place at two levels: mRNA translation inhibition or mRNA degradation [54], the former leading to the latter. Each miRNA has multiple mRNA targets and each mRNA can be the target of multiple miRNAs [55]. There is therefore a multiple control at the level of both miRNAs and mRNAs (Figure 1A) [55, 56]. Thus any change in miRNA expression may lead to many slight changes of targeted mRNAs. Furthermore, each mRNA may be the target of several miRNA variations leading eventually to major quantitative changes of mRNA expression. Also, slight effects of one miRNA on several mRNAs and their derived proteins of a specific pathway may lead, by multiplying these effects, to sizeable changes in the signaling cascade [57]. Multiple redundant pathways and feedback loops further complicate this analysis. Moreover, the action of a specific miRNA may be blocked due to the increased presence of RNAs containing the targeted sequence (“sponge” RNAs). This can produce a “dilution” of the repression function of the miRNA on its conventional mRNA target(s) [58]. Consequently, this complex regulation network should be considered when characterizing the function of a given miRNA. It is hazardous to resume the action of one miRNA to the inhibition of one or several mRNA targets even if these mRNAs showed modulated expressions (Figure 1B). Multiple cross-linked pathways represent a more realistic representation of the true molecular impacts of miRNAs but this is rarely explored.

#### miRNAs could act as long term mRNA expression regulators

A further pitfall in the analysis of miRNA expression data is the assumption that these account for most changes in mRNA expression. In fact major changes in mRNA expression occur, especially on a short-term basis, at the level of transcription and independently of the miRNAs. Transcription or half-life of any mRNA is controlled at many different levels. For instance long-term TSH or EGF/serum treatments of cultured human thyroid cells are able to mimic autonomous adenoma or PTC respectively, in terms of mRNA expression. However, no major change is observed for miRNAs, suggesting that the mRNA regulations induced by these physiological agents occur independently [59]. Similarly, in autonomous adenoma only 4 of the 841 investigated miRNA *vs* 483 of the 22000 mRNA are modulated between the tumor and the normal cells, by a factor greater than |2| [60]. Autonomous adenoma is a hyperfunctioning tumor presenting qualitatively normal follicular cells. The thyroid function is not qualitatively modified but the level of activity of the tissue is enhanced and mRNA expression is quantitatively modified, unlike miRNA. On the other hand, PTC shows qualitative alterations and up to 120 miRNAs and almost half of the mRNA expressions were found modulated between cancers and normal samples [15, 61].

These caveats on the role of miRNAs are also in line with the observations upon *in vivo* Dicer inactivation in thyroid. In mouse models, the arrest of mature miRNAs generation induces progressive loss of function and cell dedifferentiation but increases cell proliferation only after a long time. Moreover, this increase seems inefficient and might be limited by the activation of the TGF-β pathway or others mechanisms that have to be characterized. Indeed, these mice do not show an increased thyroid size or nodules [62–64]. Interestingly, the inducible arrest of mature miRNAs generation in adult mice leads to progressive cell dedifferentiation, increased cell proliferation but no loss of thyroid function. Again, no variation of the thyroid size and no nodule were reported [64]. By contrast, the rare human Dicer inactivated syndrome is associated with an increased risk of hyperplasic multi-nodular goiters. This syndrome predisposes to different types of tumors leading in rare cases to PTC development [65, 66]. Despite the differences observed between human and mouse models, these studies commonly show that Dicer is required for the long term maintenance of thyroid follicular organization and thyrocyte differentiation. Therefore, the role of miRNAs is mainly “the precision in the orchestration” [58] or insuring a stabilization of the steady state [67] and control of expression noise [68].

#### Identification of mRNA targets

Multiple computational programs have been developed for the identification of mRNA targets of miRNAs but often these do not provide coherent lists [69]. While one program may suggest hundreds of possible targets, combining two programs reduces the list to only a few common possible targets and three programs might show no overlap at all. This leads to the use of the common list resulting from only two programs. The microarrays and deep sequencing datasets of miRNA and mRNA expressions could be used together, with prediction targets programs, to create new global bioinformatics analyses. This methodology would highlight the most interesting mRNA target - miRNA couples and makes pathways analyses easier. Many algorithms allowing those analyses have been proposed recently and could be used in the future [70–73].

### Functions of miRNAs: descriptions and analyses

The above considerations could lead to a skeptical appraisal of the studies investigating in depth one specific miRNA in thyroid carcinomas, its target(s) inhibition and the validation of this interaction by using cell lines. We performed an exhaustive review of the literature considering only studies that showed or mentioned a proof of direct interaction (e.g. luciferase assays) between the considered miRNA and its proposed mRNA target(s). We therefore referenced information regarding 44 different miRNAs (supplementary file 2). Most of these miRNAs are presented as potential major elements in the biology of the tumor involved. However, only 9 miRNAs are common to several studies. Five of them are well characterized for their involvement in many other cancer types: miR-21-5p, miR-145-5p, miR-221-3p, miR-222-3p and miR-101-3p [74]. Most of the up-regulated miRNAs in PTC, FTC or ATC are described as proto-oncogenic miRNAs (oncomiR) and most of the down-regulated miRNAs are described as tumor suppressors. Nevertheless, some studies suggest that the modulation of the studied miRNAs could be due to a cell response against the transformation process. This has been described for miR-20a-5p [75], miR-195-5p [76], miR-112-5p [77] and both miR-146a-5p and miR-146b-5p [78, 79]. Interestingly, knockout mice for miR-146a gene (which has been described as overexpressed in PTC and ATC by some studies) develop early hyperplasia in prostate, followed by the appearance of internal prostatic intraepithelial neoplasia in older mice [80]. Similarly, old miR-146a knockout mice show myeloid sarcomas and lymphomas [81]. These data suggest that miR-146a-5p is a tumor suppressor miRNA, rather than an oncomiR, that has a protective role against tumor initiation. Other miRNAs described here could have a similar role.

The luciferase assay is the standard *in vitro* assay used to study the direct interaction between a miRNA and its targeted mRNA. It has been shown that this assay may present variations depending on the cell line used [82]. However, only half of the studies reported (supplementary file 2) performed or referenced luciferase assays on human thyroid carcinoma cell lines. Therefore, further validation is required for the remaining studies regarding the direct inhibition of the targeted mRNA(s). Furthermore, our group has shown that the most commonly used human thyroid cancer cell lines (BCPAP, TPC-1, K1, FTC133, WRO and 8505C) actually present similar mRNA and miRNA expression profiles. Our studies suggest that these cell lines are indeed better models for ATC even if they originated from PTC or FTC [59, 83]. Nevertheless, this fact is rarely considered, and these cells are frequently used in *in vitro* miRNA functional assays and Xenograft mouse models.

Most of the referenced studies designed their research strategies considering the dogmatic linear model of one miRNA - one mRNA (Figure 1). Their methodologies did not consider the whole spectrum of action of the studied miRNAs and they presented some of the problems previously mentioned. This leads to discrepancies in the literature. From our review, we can draw three explicit examples:

#### miR-21-5p

different functional studies showed that miR-21-5p inhibits thyroid differentiation but also increases cell proliferation and invasion. To explain these results, targeted mRNAs such as THRB, PTEN and PDCD4 have been reported as repressed. However, the direct inhibition of these mRNAs by miR-21-5p has only been proved in non-thyroid cell lines [84–87]. On the other hand, Frezzetti *et al.* [88] performed target and pathway bioinformatics analyses based on the modulated mRNAs following an *in vitro* increase of miR-21-5p expression in thyroid cells. Down-regulated mRNAs associated with the up-regulation of miR-21-5p are mainly involved in the cell cycle which is composed by validated targets, predicted targets of miR-21-5p but also indirect targets of this miRNA such as p53 and cyclin B1. This unconventional but more realistic methodology revealed the potential major effect of miR-21-5p in a follicular context. This effect is mainly related to the inhibition of the cell cycle and could be less related to cell invasion or dedifferentiation.

#### miR-146a-5p

this miRNA was first presented as an oncomiR able to protect ATC cell lines against chemotherapeutic drug-induced apoptosis [89]. However, it has been shown more recently that this miRNA is able to decrease cell proliferation and increase cell apoptosis in human PTC cell lines by targeting PKCε [78]. This suggests a dual role for this miRNA in thyroid cancer progression.

#### miR-146b-5p

this miRNA is one of the strongest overexpressed miRNA in PTC. It has been described first as an oncomiR which increases cell proliferation by targeting SMAD 4 but maintains the cells in a differentiated state and protects them from the TGF-β anti-proliferative signal [90]. However, TGF-β has a dual role in epithelial cancer progression. It has been shown that miR-146b-5p is up-regulated when inducing EMT *in vitro* by long term TGF-β treatment and this miRNA may have an inhibitory role on cell proliferation and invasion [79]. Recently, using similar methodologies, miR-146b-5p has been described to promote cell invasion, induce EMT and PTC metastasis through the activation of the Wnt/β-catenin signaling pathway [91]. The three mentioned studies inhibited miR-146b-5p expression by anti-miR siRNA in TPC1 or K1 cells but described opposite results regarding cell proliferation or cell invasion [79, 90, 91]. These three examples clearly show the necessity of a multi-targets approach to characterize the physiological function of a miRNA.

**Figure 2:**
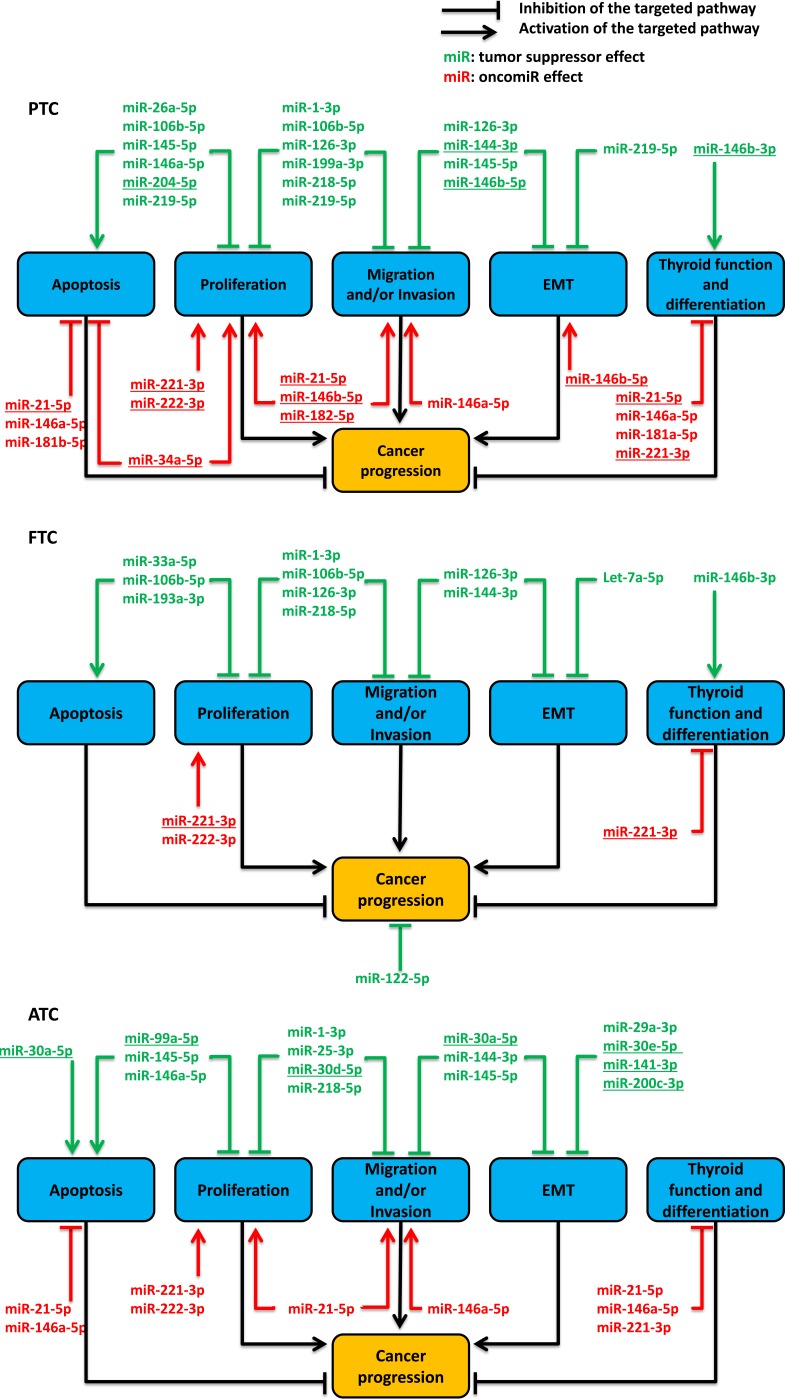
synthesis of described functions of miRNAs in the non-medullary thyroid carcinomas tumorigenesis following literature analysis, until October 2015 We did not consider studies which reported a modulation not observed in at least one of our referenced general miRNA expression profile studies on *in vivo* samples. PTC: papillary thyroid cancer; FTC: follicular thyroid cancer; ATC: anaplastic thyroid cancer; EMT: epithelial to mesenchymal transition. The underlined miRNAs are the commonly reported modulated miRNAs in the general expression profiles studies (Table 4). Therefore, they are the most relevant miRNAs involved in thyroid carcinomas development.

We summarized the function proposed for the considered miRNAs in the three non-medullary thyroid carcinoma types. We excluded studies reporting a modulation never observed in at least one of our referenced 24 general miRNA expression profile studies (supplementary file 1). Therefore, we used 35 of the 44 referenced miRNAs (Figure 2 and supplementary file 2). Five main and interconnected cell functions were analyzed across the different studies: apoptosis, proliferation, migration and invasion, EMT, differentiation and thyroid function, but mainly with *in vitro* experiments. Most of the characterized miRNAs showed multiple actions on multiple pathways. We found approximately the same number of functional studies describing either oncomiRs or tumor suppressor miRNAs in PTC. Unlike oncomiRs, the list of tumor suppressor miRNAs analyzed shows little similarity with the list of commonly reported modulated miRNAs in the small RNA deep sequencing studies (Figure 2 and Table 4). FTC functional studies mainly described tumor suppressor miRNAs and a few oncomiRs. However, only miR-221-3p is commonly found modulated in the general miRNA expression profile studies (Figure 2 and Table 4). ATC functional studies described tumor suppressor miRNAs and oncomiRs but only the oncomiRs are common with PTC or FTC studies. In addition, only a few tumor suppressor miRNAs are commonly reported under-expressed in the general miRNA expression profile studies (Figure 2 and Table 4).

**Table 4:** commonly reported modulated miRNAs in non-medullary thyroid carcinomas compared to normal tissues

	Up-regulated	Down-regulated
PTC μA and qRT-PCR	miR-146b-5p, miR-221-3p and miR-222-3p (miR-21-5p, miR-31-5p, miR-34a-5p, miR-146b-5p, miR-181b-5p, miR-221-3p, miR-222-3p, miR-224-5p, miR-375, miR-551b-3p)*	(miR-7-5p, miR-138-5p)*
PTC deepSeq	miR-21-3p, miR-21-5p,miR-31-3p, miR-31-5p,miR-34a-5p, miR-146b-3p, miR-146b-5p, miR-182-5p, miR-183-5p, miR-221-3p, miR-221-5p, miR-222-3p, miR-551b-3p	miR-7-2-3p, miR-30a-3p,miR-100-5p, miR-138-3p, miR-138-5p, miR-139-5p, miR-144-3p, miR-144-5p, miR-152-3p, miR-204-5p, miR-451a, miR-486-3p, miR-486-5p, miR-652-3p, miR-873-5p, miR-874-3p, miR-1179
FTC whole	miR-96-5p, miR-182-5p miR-221-3p (miR-183-5p, miR-222-3p)*	(miR-31-5p, miR-199a-5p, miR-199b-5p)*
ATC μA		let-7f-5p, let-7g-5p, miR-26a-5p, miR-26b-3p, miR-30d-5p, miR-30e-5p, miR-30a-5p, miR-99a-5p, miR-99b-5p, miR-125a-5p, miR-125b-5p, miR-135a-5p, miR-138-5p, miR-141-3p miR-200b-3p

Referenced miRNAs are modulated in the majority of the expression profiles studies described in Tables 1 to 3.

* miRNAs in brackets are modulated in at least three different expression profiles studies. The considered studies for each tumor type are mentioned: μA: miRNA microarray profiling studies; qRT-PCR: quantitative PCR profiling studies; deepSeq: small RNA deep sequencing profiling studies; whole: all methodologies.

Some studied miRNAs (e.g. miR-1-3p, miR-106b-5p and miR-144-3p) have been described as modulated in only one or a few of the general miRNA expression profile studies (supplementary file 1). Nevertheless, functional experiments reported an effect of these miRNAs. This could be explained by many issues such as the incorrect molecular representativeness of the cell line used, the non-physiological *in vitro* experimental procedure, the differences in the microarray platform used in the different studies, the absence of multiple small RNA deep-sequencing studies for FTC and ATC, etc. We showed in this review (point 2) that PTC and FTC samples present common overexpressed miRNAs: miR-182-5p, miR-183-5p, miR-221-3p and miR-222-3p. These miRNAs have all been characterized as activators of thyrocytes proliferation [92–95]. In addition, miR-182-5p and miR-183-5p increase cell invasion [93, 95]. The modulation of these miRNAs may therefore represent a common pathological feature in differentiated thyroid carcinomas. For instance, some validated targets of these miRNAs (e.g. PTEN, PDCD4, p27) are involved in the negative regulation of the PI3K/Akt pathway which has been described as activated at different levels in thyroid carcinomas [96]. miR-221-3p and miR-222-3p are up-regulated in invasive PTC compared to non-invasive PTC [97, 98] and show increased expressions in BRAF V600E mutated PTC samples [15]. In accordance, this mutation has been associated with an increased aggressiveness of PTC [99, 100]. Moreover, miR-222-3p is up-regulated in half of the general miRNA expression profile studies on ATC samples (supplementary file 1). On the other hand, PTC and FTC samples exhibit large differences in their miRNA expression and functional profiles. Thus, the distinction of these two types of carcinoma on a pathological basis supported already by clinical features and by a different set of oncogenic mutations [8] corresponds clearly to a gene expression phenotype. Surprisingly, ATC samples, which may derive from PTC and FTC, exhibit a widely different miRNA expression pattern. Notably, some miRNA families that have been described as EMT and cancer progression inhibitors are down-regulated (e;g miR-30 or miR-200 families) [53, 101]. This dramatic shift in miRNA expression corresponds to a similar shift in pathological and clinical phenotypes. This could explain the extraordinary invasive capacity of this cancer. As one of the effects of p53 is to induce miRNA expression [102–104], this could be the result of a p53 inactivation. The general down-regulation of miRNAs could represent the final common pathway leading to ATC in response to various mechanisms including and besides p53 inactivation. In accordance, inactivating mutations of p53 are found in 55% of ATC [105] and are sufficient to confer ATC properties to PI3K activated thyroid cells in a mouse model [106].

### Conclusions, perspectives and remaining challenges

Even if the meaning and function of individual changes in miRNA expression thyroid carcinomas are difficult to interpret, there is no doubt that their general pattern modification is meaningful. However, the detailed correlation of gene expression with the pathological and clinical phenotypes remains to be done. So far, the list of commonly modulated miRNAs (Table 4) shows low overlaps with the list of studied functional miRNAs (Figure 2) but allows the identification of the most interesting miRNAs that could be directly involved in thyroid cancer tumorigenesis. Such miRNAs are miR-21-5p, miR-34a-5p, miR-144-3p, miR-146b-3p, miR-146b-5p, miR-182-5p, miR-204-5p, miR-221-3p and miR-222-3p for PTC; miR-30a-5p, miR-30d-5p, miR-30e-5p, miR-99a-5p, miR-141-3p, miR-200c-3p for ATC (Figure 2). Nevertheless, only miR-221-3p shows both functional and largely confirmed expression data which support its potential direct involvement in FTC tumorigenesis (Figure 2). Recently, cell adhesion pathways have been described as deeply modulated in PTC [18]. So far, these pathways are poorly analyzed in functional studies.

The dogmatic linear control model of one miRNA - one mRNA is so prevalent in the literature that the functional results must be interpreted with care and validated in different studies. It is quite likely that some of the described miRNAs may have an important role in thyroid carcinomas tumorigenesis and/or progression but this remains to be confirmed. The functional characterizations reported are limited by the weak use of *in vivo* models. Furthermore, the Xenograft mice models are technically simple, reproducible and cost effective but present some disadvantages that could be managed by the use of other *in vivo* models. For instance, orthotopic tumor mouse models have been developed by injections of cell lines in the appropriate anatomic sites [107, 108]. These models are more cumbersome to develop but allow tumor growth in the correct microenvironment and the development of tumor metastasis through the same anatomical routes as the original tumor, improving functional characterization [109]. However, none of the studies reported in our review used such models. Moreover, classical oncogenes or tumor suppressor genes are characterized by the generation of tumors in transgenic mice. This model is more difficult to develop but present multiple advantages. For instance, the studied gene can be characterized *in situ,* in mice with a functional immune system and it does not require cell lines. However, to our knowledge, no thyroid tumor has so far been generated in transgenic mice with a modulation of expression of one or several miRNAs. This would be convincing and would represent a great progress in the field. Although “driver” modulations of expression could be found, as it is the case for the increased expression of miR-155 in lymphoid cancer in mice [110], most of the miRNA modulations could also be considered as passenger phenomena. Interestingly, transgenic mouse models of some of the miRNAs that could be directly involved in thyroid cancer tumorigenesis have been developed in other organs. Particularly, a mouse model exhibiting an overexpression of miR-221-3p in the liver has been developed. This model shows spontaneous nodular liver lesions and an acceleration of tumor development, defining this miRNA as an important oncomiR in the hepatocellular carcinoma [111]. This miRNA should be considered for the future development of transgenic mouse models of thyroid cancers.

The studied miRNAs are often proposed as therapeutic targets. The utility of miRNAs or miRNAs inhibitors as therapeutic tools is a recent and very interesting field of research already showing promising results [112]. However, to our knowledge, no miRNA has been described and validated yet as a clinical therapeutic agent or target for thyroid carcinomas. This would greatly improve the treatment especially for the lethal ATC. The correct delivery of a drug to the targeted tissue and simultaneously weak toxicity on other tissues is a general challenge in cancer therapy and thus in miRNA therapy as well. Mimic miRNAs or inhibitors of miRNAs could be delivered through different techniques. So far, the safety and the efficacy of miRNA therapy have been proven in different *in vivo* studies [112, 113]. The absence of systematic toxicity can simply be defined by the absence of obvious cytotoxicity in more important organs [114]. Nevertheless, other methods have been established, such as the inclusion of miRNA target sites within the 3′ UTR of E1A gene, essential for the viral replication of oncolytic adenovirus. This leads to a specific lysis in cells which do not express the miRNA. This approach has been used, *in vitro* and in mouse models, to target hepatocellular carcinomas cells because these do not express miR-199, unlike normal liver cells and other tissues [115]. This approach could be combined with other strategies: for instance, the addition of anti-oncomiR genes in the genome of this virus could improve the regression of the hepatocellular carcinomas [116].

### Recommendations

Clearly, thyroid carcinomas are not the only tumors analyzed for miRNA expression and function. Therefore, we propose several controls that could minimize to some extent the described drawbacks and allow distinguishing important switches from the noise affecting miRNA expression in each cancer type:

- investigate most frequent and quantitatively important changes in expression, being aware that different cell populations may coexist in the studied surgical sample;
- test the effects of miRNAs in different models (e.g. cell lines) and not only in the most responsive one;
- use in priority the most representative cell lines which are derived from the cancer of interest (even for the luciferase assay), consider the different *in vivo* models;
- always test the effects of the miRNA on the whole mRNA population and not only on the supposed targets, being aware that the miRNA could have both direct and indirect effects on multiple pathways;
- consider other published studies on the same miRNA in other systems and analyze the targets and pathways proposed.

## GENERAL CONCLUSIONS

The research on miRNA expression signatures in thyroid non-medullary carcinomas using a suitable combination of findings from several studies has some future. The appearance of small RNA deep sequencing data will increase the efficiency of diagnostic signature and will be the starting point of new functional studies.

In physiopathology, the main challenge is to distinguish minor and major contributions to the phenotype and consequently mRNA targets. Concluding that one miRNA may be a driver of tumorigenesis requires rigorous proofs. Future studies have to consider and to determine the exact regulatory network between miRNAs and mRNAs. The field of therapeutic options is wide open but the possibility of interfering with other organs while treating thyroid cancer must be carefully evaluated.

The extension of these conclusions on other cancer types deserves to be considered.

## SUPPLEMENTARY FILES AND TABLES




