# Supplementary material for: miRNA expression and function in thyroid carcinomas: a comparative and critical analysis and a model for other cancers
Source: Oncotarget. 2016 May 28;7(32):52475–92. doi: 10.18632/oncotarget.9655 (PMC5239568; doi:10.18632/oncotarget.9655)
Supplement: Supplementary file 1 [file oncotarget-07-52475-s001.pdf]

miRNA	Cancer types	Modulation	Targeted mRNAs	Pathways involved	References	Luciferase assay
**let-7a-5p**	FA, FTC	↓	FXYD5	↑EMT	Colamaio *et al.* 2012 [124]	human megakaryoblastic leukemia cell line: MEG-01
**miR-1-3p**	PTC, FTC, ATC	↓	CCND2, CXCR4, SDF-1α	↑cell proliferation and cell migration and invasion	Leone *et al.* 2011 [125]	human thyroid carcinoma cell line : FRO
**miR-21-5p**	PTC, ATC	↑	THRB<br><br>PDCD4, PTEN	↓thyroid function<br><br>↑cell growth (*in vivo*)<br>↑cell proliferation and invasion<br>↓cell apoptosis and differentiation	Jazdzewski *et al.* 2011 [85]<br><br>Talotta *et al.* 2009 [84]<br>Frezzetti *et al.* 2011 * [88]<br>Zhang *et al.* 2014 [86]<br>Haghpanah *et al.* 2015[87]<br>*Frankel et al. 2008 [82]*<br>*Lu et al. 2008 [126]*<br><br>*Asangani et al. 2008 [127]*	human embryonic kidney cell line: HEK 293<br>/<br>/<br>/<br>/<br>human breast carcinoma cell line: MCF7<br>human embryonic kidney cell line: HEK 293<br>human colorectal cancer cell lines: Colo206F, RKO and Hct116
**miR-25-3p**	ATC	↓	EZH2	↑cell proliferation and migration	Esposito *et al.* 2012 [128]	human thyroid carcinoma cell line: ACT-1
**miR-26a-5p**	PTC	↓	CKS2	↑Cell proliferation (*in vivo*)<br>↓cell apoptosis (*in vivo*)	Lv *et al.* 2013 * [129]	human embryonic kidney cell line: HEK 293
**miR-29a-3p**	ATC	↓	LOX	↑EMT	Hébrant *et al.* 2014 [53]	human embryonic kidney cell line: HEK 293
**miR-30a-5p**	ATC	↓	LOX	↑cell growth<br>↑metastasis formation (*in vivo*)<br>↓cell apoptosis	Boufraqech *et al.** 2015 [130]	human thyroid carcinoma cell line: THJ-16T
**miR-30d-5p**	ATC	↓	EZH2<br><br>BECN1	↑cell proliferation and migration<br><br>↑Autophagy (*in vivo*)	Esposito *et al.* 2012 [128]<br><br>Zhang *et al.* 2014 * [131]	human thyroid carcinoma cell line: ACT-1<br>human thyroid carcinoma cell lines: SW1736 and 8305C
**miR-30e-5p**	ATC	↓	ZEB2, SMAD2	↑EMT	Braun *et al.* 2010 [101]	*In vitro* ATC derived cells

**miR-33a-5p**	FTC	↓	MYC	↑cell proliferation	Takeshita *et al.* 2013 [132]	human thyroid carcinoma cell line: WRO
**miR-34a-5p**	PTC	↑	GAS1	↑cell proliferation<br>↓cell apoptosis	Ma *et al.* 2013 [133]	human thyroid carcinoma cell line: TPC1
**miR-99a-5p**	ATC	↓	mTOR	↑cell growth (*in vivo*)<br>↑Cell proliferation<br>↓cell apoptosis	Huang *et al.* 2015 * [134]	human embryonic kidney cell line: HEK 293
**miR-106b-5p**	PTC, FTC	↓	C1orf24	↑cell cycle and migration<br>↓cell apoptosis	Carvalheira *et al.* 2015 [135]	rat thyroid follicular cell line: PCCl3
**miR-122-5p**	FTC	↑	ADAM17	↑Antiangiogenic pathways (*in vivo*)	Reddi *et al.* 2011 * [77]<br>Tsai *et al.* 2009 [136]	/<br>human embryonic kidney cell line: HEK 293
**miR-126-3p**	PTC, FTC	↓	SLC7A5, ADAM9	↑cell growth, migration and metastasis formation (*in vivo*)	Xiong *et al.* 2015 * [137]	human thyroid carcinoma cell line: FTC-133
**miR-138-5p**	ATC	↓	hTERT		Mitomo *et al.* 2008 [138]	human embryonic kidney cell line: HEK 293
**miR-141-3p**	ATC	↓	TGFBR1, SMAD2	↑EMT	Braun *et al.* 2010 [101]	*In vitro* ATC derived cells
**miR-144-3p**	PTC, FTC, ATC	↓	ZEB1 and ZEB2	↑cell invasion and migration (EMT)	Guan *et al.*2015 [139]	human thyroid carcinoma cell lines: K1 and WRO
**miR-145-5p**	PTC, ATC	↓	AKT3<br><br>DUSP6	↑cell proliferation (*in vivo*)<br>↑metastasis formation (*in vivo*)<br>↓cell apoptosis<br><br>↑cell growth	Boufraqech *et al.* * 2014 [140]<br><br>Gu *et al.* 2015 [141]	human thyroid carcinoma cell lines: FTC-133, TPC-1, 8505c<br><br>human embryonic kidney cell line: HEK 293
**miR-146a-5p**	PTC, ATC	↑	IRAK1, TRAF6, CCDC6<br><br>THRB	↓gene Toll-like receptor and cytokine signalling pathway<br>↑cell invasion<br>↓cell apoptosis<br><br>↓thyroid function	Jazdzewski *et al.* 2008 [142]<br>Pacifico *et al.*2010 [89]<br><br>Jazdzewski *et al.* 2011 [85]	human osteosarcoma cell line: U2OS<br>human thyroid carcinoma cell line: FRO<br><br>human embryonic kidney cell line: HEK 293

			PRKCE	↓cell proliferation (*in vivo*)<br>↑cell apoptosis	Zhang *et al.* 2014 * [78]	human prostate epithelial cell line: RWPE-1
**miR-146b-3p**	PTC, PDFTC	↑	NIS, PAX8	↓thyroid function<br>↓cell differentiation	Li *et al.* 2015 [143]<br>Riesco-Eizaguirre *et al.* 2015 [18]	human thyroid carcinoma cell line: FTC-133<br>human adenocarcinoma cell line: HeLa
**miR-146b-5p**	PTC	↑	SMAD4<br><br>ZNRF3	↑cell proliferation<br><br>↑migration and invasion<br>↑EMT	Geraldo *et al.* 2012 [90]<br><br>Deng *et al.* 2015 [91]	human thyroid carcinoma cell line : ARO<br><br>human thyroid carcinoma cell lines: K1 and TPC-1
**miR-155-5p**	PTC	↑	APC	↑cell growth (*in vitro*)	Zhang *et al.* 2013 * [144]	human thyroid carcinoma cell lines: TPC-1 and CGTH-W3
**miR-181a-5p**	PTC	↑	THRB	↓thyroid function	Jazdzewski *et al.* 2011 [85]	human embryonic kidney cell line: HEK 293
**miR-181b-5p**	PTC	↑	CYLD	↑cell growth<br>↓cell apoptosis	Li *et al.*2014 [145]	human embryonic kidney cell line: HEK 293
**miR-182-5p**	PTC	↑	CHL1	↑cell proliferation and invasion (*in vivo*)	Zhu *et al.* 2014 * [93]	human thyroid carcinoma cell lines: TPC-1 and BCPAP
**miR-183-5p**	PTC	↑	PDCD4	↑cell proliferation, migration and invasion<br>↓cell apoptosis	Wei *et al.* 2015 [95]	human thyroid carcinoma cell lines: TPC-1
**miR-193a-3p**	FTC	↓	SLC7A5	↑cell proliferation<br>↓cell apoptosis	Takeshita *et al.* 2013 [132]	human thyroid carcinoma cell line: WRO
**miR-199a-3p**	PTC	↓	MET, mTOR	↑cell proliferation and migration<br>↓macropinocytosis pathway	Minna *et al.* 2014 [146]<br>*Migliore et al. 2008 [147]*<br>*Fornari et al. 2010 [148]*	/<br>African green monkey kidney fibroblast like cell line: cos-7<br>Human hepatocellular carcinoma cell lines:Huh-7 and SNU475

**miR-200c-3p**	ATC	↓	SMAD2	↑EMT	Braun *et al.* 2010 [101]	*In vitro* ATC derived cells
**miR-204-5p**	PTC	↓	IGFBP5	↑cell growth (*in vivo*)<br>↑cell proliferation<br>↓cell apoptosis	Liu *et al.* 2015 * [149]	human embryonic kidney cell line: HEK 293
**miR-218-5p**	PTC, FTC, ATC	↓	PDGFRA , PLCG1	↑cell growth (*in vivo*)<br>↑cell proliferation, migration and invasion	Guan *et al.* 2013 * [150]	human thyroid carcinoma cell lines: WRO and SW579
**miR-219-5p**	PTC	↓	ESR1	↑cell proliferation and migration<br>↓cell apoptosis	Huang *et al.* 2014 [151]	human thyroid carcinoma cell line: K1
**miR-221-3p**	PTC, FTC, ATC	↑	p27^{Kip1}(CDKN1B)<br>THRB<br>PTEN	↑cell cycle<br>↓thyroid function<br>↑cell growth	Visone *et al.* 2007 [92]<br>Jazdzewski *et al.* 2011 [85]<br>Mardente *et al.* 2015 [94]<br>*Chun-zhi et al. 2010 [152]*	human thyroid carcinoma cell line: TPC1 and human adenocarcinoma cell line: HeLa<br>human embryonic kidney cell line: HEK 293<br>/<br>human gastric cancer cell line: SGC7901
**miR-222-3p**	PTC, FTC, ATC	↑	p27^{Kip1}(CDKN1B)<br>PTEN	↑cell cycle<br>↑cell growth	Visone *et al.* 2007 [92]<br>Mardente *et al.* 2015 [94]<br>*Chun-zhi et al. 2010 [152]*	human thyroid carcinoma cell line: TPC1 and human adenocarcinoma cell line: HeLa<br>/<br>human gastric cancer cell line: SGC7901
**miR-20a-5p**	ATC	↑	LIMK1	↓cell growth (in vivo) and invasion	Xiong *et al.* 2014 * [75]	human thyroid carcinoma cell line: FTC-133
**miR-101-3p**	PTC	↓	RAC1	↑cell proliferation, migration and invasion	Lin *et al.* 2014 [153]<br>Wang et al. 2014 [154]	human thyroid carcinoma cell line: K1<br>human embryonic kidney cell line: HEK 293
**miR-149-5p**	FTC	↓	FOSL1	↑cell proliferation	Takeshita *et al.* 2013 [132]	human thyroid carcinoma cell line: WRO
**miR-195-5p**	PTC	↓	ZNF367	↓cell invasion	Jain *et al.* 2014 [76]	human adrenal carcinoma cell line:

						SW13
**miR-205-5p**	PTC	↓	VEGF-A	↑cell proliferation<br>↓cell apoptosis and angiogenesis	Salajegheh *et al.* 2015 [155]<br>*Wu et al. 2009 [156]*	/<br><br>human embryonic kidney cell line: HEK 293
**miR-449b-5p**	PTC,FTC,ATC	↓	MET	↑cell proliferation	Chen *et al.* 2015 [157]	human thyroid carcinoma cell line: TT
**miR-539-5p**	PTC, FTC	↓	CARMA1	↑cell growth, migration and invasion	Gu and Sun 2015 [158]	human thyroid carcinoma cell line: K1
**miR-618**	ATC	↓	XIAP	↑cell proliferation, invasion and migration<br>↓cell apoptosis	Cheng *et al.* 2014 [159]	Human thyroid call line: Nthy-ori 3-1
**miR-886-3p**	PTC	↓	CCDC6	↑cell proliferation and migration	Xiong *et al.* 2011 [160]	human thyroid carcinoma cell line: TPC-1

Supplementary file 2

Synthesis of miRNA functional studies of non-medullary thyroid carcinomas following literature analysis, until October 2015: The modulation of the studied miRNA, the targeted mRNA(s) described, the pathway(s) analysed and the cell line(s) used to show the direct interaction between the miRNA and its mRNA target are referenced. We only considered studies which showed or mentioned a proof of direct interaction (Luciferase assays) between the considered miRNA and its hypothetical mRNA target(s), until October 2015. When no luciferase assay was performed, the reference used by the authors to assume the direct interaction is provided in italic. The double black border separates the studies which are in accordance with at least one general miRNA expression profile study described in supplementary file 1 (upper part of the table) and those that are not in accordance (lower part). The term "cell growth" refers to analyses showing an effect on cell survival without providing cell proliferation and/or apoptosis data. The term "*in vivo*" refers to modulations of pathways that were confirmed in Xenograft mouse models. PTC: papillary thyroid carcinoma; FTC: follicular thyroid carcinoma; ATC: anaplastic thyroid carcinoma; modulation ↑ or ↓: miRNA respectively up-regulated or down-regulated in tumors compared to normal samples in the study; pathway ↑ or ↓: consequence of the modulation of the miRNA on the targeted pathway; /: no luciferase assay performed; *: study which performed *in vivo* functional analyses.

FXYD5: dysadherin; CCND2: cyclin D2; CXCR4: C-X-C chemokine receptor type 4; SDF-1α: stromal cell derive fatocr 1; THRB: thyroid hormone receptor β; PDCD4: programmed cell death 4; PTEN: phosphatase and tensin homolog; EZH2: polycomb protein enhancer of zeste 2; CKS2: CDC28 protein kinase regulatory subunit 2; LOX: lysyl oxidase; BECN1: beclin 1; ZEB2: zinc finger E-box binding homeobox 2; MYC: myelocytomatosis viral oncogene homolog; GAS1: growth arrest specific1; MET: MET proto-oncogene, receptor tyrosine kinase; mTOR: mechanistic target of rapamycin; C1orf24: family with sequence similarity 129, member A; ADAM17: ADAM metallopeptidase domain 17; SLC7A5: carrier family 7 member 5; ADAM9: ADAM metallopeptidase domain 9; TGFBR1: transforming growth factor, beta receptor 1; SMAD2: SMAD family member 2; ZEB1: zinc finger E-box binding homeobox 1; AKT3: v-akt murine thymoma viral oncogene homolog 3; hTERT: human telomerase reverse transcriptase; IRAK1: IL-1 receptor-associated kinase 1; TRAF6: TNF receptor-associated factor 6; CCDC6: the papillary thyroid carcinoma 1 gene; PRKCE: protein kinase C, epsilon; NIS: solute carrier family 5 member 5; PAX8: paired box 8; SMAD4: mothers against decapentaplegic homolog 4; ZNRF3: zinc and ring finger 3; APC: adenomatous polyposis coli; DUSP6: dual specificity phosphatase 6; CYLD: cylindromatosis; CHL1: cell adhesion molecule L1-like; IGFBP5: insulin-like growth factor-binding protein 5; PDGFRA: platelet-derived growth factor receptor, alpha polypeptide; PLCG1: phospholipase C, gamma 1; ESR1: estrogen receptor 1; $p27^{Kip1}$(CDKN1B): cyclin-dependent kinase inhibitor 1B; LIMK1: LIM domain kinase 1; RAC1: ras-related C3 botulinum toxin substrate 1; FOSL1: fos-related antigen 1; ZNF367: zinc finger protein 367; VEGF-A: vascular endothelial growth factor A; or CARMA1: caspase recruitment domain family, member 11; XIAP: X-linked inhibitor of apoptosis protein; CDC6: cell division cycle 6.

References

124. Colamaio M, Calì G, Sarnataro D, Borbone E, Pallante P, Decaussin-Petrucci M, Nitsch L, Croce CM, Battista S, and Fusco A. Let-7a down-regulation plays a role in thyroid neoplasias of follicular histotype affecting cell adhesion and migration through its ability to target the FXYD5 (dysadherin) gene. The Journal of Clinical Endocrinolgy and Metabolism. 2012*;* 97: 2168–2178. doi: 10.1210/jc.2012-1929

125. Leone V, D'Angelo D, Rubio I, de Freitas PM, Federico A, Colamaio M, Pallante P, Medeiros-Neto G, and Fusco A. MiR-1 is a tumor suppressor in thyroid carcinogenesis targeting CCND2, CXCR4, and SDF-1alpha. The Journal of Clinical Endocrinolgy. 2011; 96: 1388–1398. doi: 10.1210/jc.2011-0345

126. Lu Z, Liu M, Stribinskis V, Klinge CM, Ramos KS, Colburn NH, and Li Y. MicroRNA-21 promotes cell transformation by targeting the programmed cell death 4 gene. Oncogene. 2008; 27: 4373–4379. doi: 10.1038/onc.2008.72

127. Asangani IA, Rasheed SA, Nikolova DA, Leupold JH, Colburn NH, Post S, and Allgayer H. MicroRNA-21 (miR-21) post-transcriptionally downregulates tumor suppressor Pdcd4 and stimulates invasion, intravasation and metastasis in colorectal cancer. Oncogene. 2008; 27: 2128–2136.

128. Esposito F, Tornincasa M, Pallante P, Federico A, Borbone E, Pierantoni GM, and Fusco A. Down-regulation of the miR-25 and miR-30d contributes to the development of anaplastic thyroid carcinoma targeting the polycomb protein EZH2. The Journal of Clinical Endocrinolgy. 2012; 97: 710–718. doi: 10.1210/jc.2011-3068
129. Lv M, Zhang X, Li M, Chen Q, Ye M, Liang W, Ding L, Cai H, Fu D, and Lv Z. miR-26a and its Target CKS2 Modulate Cell Growth and Tumorigenesis of Papillary Thyroid Carcinoma. PLoS One. 2013; 8: 1–11. doi: 10.1371/journal.pone.0067591
130. Boufraqech M, Nilubol N, Zhang L, Gara SK, Sadowski SM, Mehta A, He M, Davis S, Dreiling J, Copland JA, Smallridge RC, Quezado MM, and Kebebew E. miR30a Inhibits LOX Expression and Anaplastic Thyroid Cancer Progression. Cancer Research. 2014; 75: 367-77. doi:10.1158/0008-5472.CAN-14-2304
131. Zhang Y, Yang WQ, Zhu H, Qian YY, Zhou L, Ren YJ, Ren XC, Zhang L, Liu XP, Liu CG, Ming ZJ, Li B, Chen B, et al. Regulation of Autophagy by miR-30d Impacts Sensitivity of Anaplastic Thyroid Carcinoma to Cisplatin. Biochemical Pharmacology. 2014; 87: 562–570. doi: 10.1016/j.bcp.2013.12.004
132. Takeshita H, Shiozaki A, Bai XH, Iitaka D, Kim H, Yang BB, Keshavjee S, and Liu M. a New Adaptor Protein, Regulates Expression of Tumor Suppressive MicroRNAs in Cancer Cells. PLoS One. 2013; 8: e59057. doi: 10.1371/journal.pone.0059057
133. Ma Y, Qin H, and Cui Y. MiR-34a targets GAS1 to promote cell proliferation and inhibit apoptosis in papillary thyroid carcinoma via PI3K/Akt/Bad pathway. Biochemical and Biophysical Research Communications.2013; 441: 958–963. doi: 10.1016/j.bbrc.2013.11.010
134. Huang H, Luo X, Wu S, and Jian B. MiR-99a Inhibits Cell Proliferation and Tumorigenesis through Targeting mTOR in Human Anaplastic Thyroid Cancer. Asian Pacific Organization for Cancer Prevention. 2015; 16: 4937–4944.
135. Carvalheira G., Nozima BH, and Cerutti JM. microRNA-106b-mediated down-regulation of C1orf24 expression induces apoptosis and suppresses invasion of thyroid cancer. Oncotarget. 2015; 6: 28357-70. doi: 10.18632/oncotarget.4947
136. Tsai WC, Hsu PW, Lai TC, Chau GY, Lin CW, Chen CM, Lin CD, Liao YL, Wang JL, Chau YP, Hsu MT, Hsiao M, Huang HD, et al. MicroRNA-122, a tumor suppressor MicroRNA that regulates intrahepatic metastasis of hepatocellular carcinoma. Hepatology. 2009; 49: 1571–1582. doi: 10.1002/hep.22806
137. Xiong Y, Kotian S, Zeiger M, Zhang L, and Kebebew E. miR-126-3p Inhibits Thyroid Cancer Cell Growth and Metastasis, and Is Associated with Aggressive Thyroid Cancer. PLoS One. 2015; 10: e0130496. doi: 10.1371/journal.pone.0130496
138. Mitomo S, Maesawa C, Ogasawara S, Iwaya T, Shibazaki M, Yashima-Abo A, Kotani K, Oikawa H, Sakurai E, Izutsu N, Kato K, Komatsu H, Ikeda K, et al. Downregulation of miR-138 is associated with overexpression of human telomerase reverse transcriptase protein in human anaplastic thyroid carcinoma cell lines. Cancer Science. 2008; 99: 280–286.
139. Guan H, Liang W, Xie Z, Li H, Liu J, Liu L, Xiu L, and Li Y. Down-regulation of miR-144 promotes thyroid cancer cell invasion by targeting ZEB1 and ZEB2. Endocrine. 2015; 48: 566–574. doi: 10.1007/s12020-014-0326-7
140. Boufraqech M, Zhang L, Jain M, Patel D, Ellis R, Xiong Y, He M, Nilubol N, Merino MJ, and Kebebew E. miR-145 suppresses thyroid cancer growth and metastasis and targets AKT3. Endocrine Relatated Cancer. 2014; 21: 517–31. doi: 10.1530/ERC-14-0077
141. Gu Y, Li D, Luo Q, Wei C, Song H, Hua K, Song J, Luo Y, Li X, and Fang L. MicroRNA-145 inhibits human papillary cancer TPC1 cell proliferation by targeting DUSP6. International Journal of Clinical and Experimental Medicine. 2015; 8: 8590–8598.
142. Jazdzewski K, Murray EL, Franssila K, Jarzab B, Schoenberg DR, and de la Chapelle A. Common SNP in pre-miR-146a decreases mature miR expression and predisposes to papillary thyroid carcinoma. Proceedings of the National Academy of Sciences. 2008; 105: 7269–74. doi:

10.1073/pnas.0802682105
143. Li L, Lv B, Chen B, Guan M, Sun Y, Li H, Zhang B, Ding C, He S, and Zeng Q. Inhibition of miR-146b expression increases radioiodine-sensitivity in poorly differential thyroid carcinoma via positively regulating NIS expression. Biochemical and Biophysical Research Communications. 2015; 462: 314–321. doi: 10.1016/j.bbrc.2015.04.134
144. Zhang X, Li M, Zuo K, Li D, Ye M, Ding L, Cai H, Fu D, Fan Y, and Lv Z. Upregulated miR-155 in papillary thyroid carcinoma promotes tumor growth by targeting APC and activating Wnt/β-catenin signaling. The Journal of Clinical Endocrinology Metabolism. 2013; 98: E1305-13. doi:10.1210/jc.2012-3602
145. Li D, Jian W, Wei C, Song H, Gu Y, Luo Y, and Fang L. Down-regulation of miR-181b promotes apoptosis by targeting CYLD in thyroid papillary cancer. International Journal of Clinical and Experimental Pathology. 2014; 7: 7672–7680.
146. Minna E, Romeo P, De Cecco L, Dugo M, Cassinelli G, Pilotti S, Degl'Innocenti D, Lanzi C, Casalini P, Pierotti MA, Greco A, and Borrello MG. miR-199a-3p displays tumor suppressor functions in papillary thyroid carcinoma. Oncotarget. 2014; 5: 2513-28.
147. Migliore C, Petrelli A, Ghiso E, Corso S, Capparuccia L, Eramo A, Comoglio PM, and Giordano S. MicroRNAs impair MET-mediated invasive growth. Cancer Research. 2008; 68: 10128-36. doi:10.1158/0008-5472.CAN-08-2148
148. Fornari F, Milazzo M, Chieco P, Negrini M, Calin GA, Grazi GL, Pollutri D, Croce CM, Bolondi L, and Gramantieri L MiR-199a-3p regulates mTOR and c-Met to influence the doxorubicin sensitivity of human hepatocarcinoma cells. Cancer Research. 2010; 70: 5184-93. doi: 10.1158/0008-5472.CAN-10-0145.
149. Liu L, Wang J, Li X, Ma J, Shi C, Zhu H, Xi Q, Zhang J, Zhao X, and Gu M.miR-204-5p suppresses cell proliferation by inhibiting IGFBP5 in papillary thyroid carcinoma. Biochemical and Biophysical Research Communications. 2015; 457: 621–6. doi: 10.1016/j.bbrc.2015.01.037
150. Guan H, Wei G, Wu J, Fang D, Liao Z, Xiao H, Li M, and Li Y. Down-regulation of miR-218-2 and its host gene SLIT3 cooperate to promote invasion and progression of thyroid cancer. The Journal of Clinical Endocrinology and Metabolism. 2013; 98: E1334-44. doi:10.1210/jc.2013-1053
151. Huang C, Cai Z, Huang M, Mao C, Zhang Q, Lin Y, Zhang X, Tang B, Chen Y, Wang X, Qian Z, Ye L, Peng Y, and Xu H. miR-219-5p Modulates Cell Growth of Papillary Thyroid Carcinoma by Targeting Estrogen Receptor α. Journal of Clinical Endocrinology and Metabolism. 2014; 100: E204-13 doi:10.1210/jc.2014-2883
152. Chun-Zhi Z, Lei H, An-Ling Z, Yan-Chao F, Xiao Y, Guang-Xiu W, Zhi-Fan J, Pei-Yu P, Qing-Yu Z, and Chun-Sheng K. MicroRNA-221 and microRNA-222 regulate gastric carcinoma cell proliferation and radioresistance by targeting PTEN. BMC Cancer. 2010; 10: 367. doi: 10.1186/1471-2407-10-367
153. Lin X, Guan H, Li H, Liu L, Liu J, Wei G, Huang Z, Liao Z, and Li Y. miR-101 inhibits cell proliferation by targeting Rac1 in papillary thyroid carcinoma. BioMedReports. 2014; 2: 122–126.
154. Wang C, Lu S, Jiang J, Jia X, Dong X, and Bu P. Hsa-microRNA-101 suppresses migration and invasion by targeting Rac1 in thyroid cancer cells. Oncology Letters. 2014; 8: 1815–1821.
155. Salajegheh A, Vosgha H, Md Rahman A, Amin M, Smith RA, and Lam AK. Modulatory role of miR-205 in angiogenesis and progression of thyroid cancer. Journal of Molecular Endocrinology. 2015; 55: 183–96. doi: 10.1530/JME-15-0182
156. Wu H, Zhu S, and Mo YY. Suppression of cell growth and invasion by miR-205 in breast cancer. Cell Research. 2009; 19: 439–448. doi: 10.1038/cr.2009.18

157. Chen L, Xu L, and Wang G. Regulation of MET-mediated proliferation of thyroid carcinoma cells by miR-449b. Tumor Biology. 2015; 36: 8653-60. doi:10.1007/s13277-015-3619-4
158. Gu L, and Sun W. MiR-539 inhibits thyroid cancer cell migration and invasion by directly targeting CARMA1. Biochemical and Biophysical Research Communications. 2015; 464:1128-33. doi:10.1016/j.bbrc.2015.07.090
159. Cheng Q, Zhang X, Xu X, and Lu X. MiR-618 inhibits anaplastic thyroid cancer by repressing XIAP in one ATC cell line. Annales d'Endocrinologie. 2014; 75: 187–93. doi: 10.1016/j.ando.2014.01.002
160. Xiong Y, Zhang L, Holloway AK, Wu X, Su L, and Kebebew E. MiR-886-3p regulates cell proliferation and migration, and is dysregulated in familial non-medullary thyroid cancer. PLoS One. 2011; 6: 1–11. doi: 10.1371/journal.pone.0024717
